# Transcervical administration of polidocanol foam prevents pregnancy in female baboons^☆^^☆☆^

**DOI:** 10.1016/j.contraception.2016.07.008

**Published:** 2016-11

**Authors:** Jeffrey T. Jensen, Carol Hanna, Shan Yao, Emily Thompson, Cassondra Bauer, Ov D. Slayden

**Affiliations:** a Department of Obstetrics & Gynecology, Oregon Health & Science University (OHSU), Portland, OR; b Division of Reproductive and Developmental Sciences, Oregon National Primate Research Center, Beaverton, OR; c Southwest National Primate Research Center, San Antonio, TX

**Keywords:** Nonhuman primate, Sterilization, Nonsurgical, Female, Polidocanol, Permanent contraception

## Abstract

**Background:**

Our objective was to conduct a pilot study to determine if transcervical administration of polidocanol foam (PF) with or without doxycycline or benzalkonium chloride (BZK) would prevent pregnancy in baboons.

**Methods:**

In study phase 1, adult cycling baboons underwent a hysterosalpingogram to evaluate tubal patency prior to transcervical infusion of 20 mL of 5% PF followed by 1 mL of saline containing 100 mg doxycycline (5%/doxy; *n*=5), 3% PF plus doxycycline (3%/doxy; *n*=4), 3% PF with 0.01% BZK (3%/BZK; *n*=4) or no additional treatment (control; *n*=9). Immediately following treatment, animals received intramuscular depot medroxyprogesterone acetate (DMPA, 2 mg/kg) to suppress cyclicity during healing and were then socially housed with males of proven fertility. The primary outcome was pregnancy within six cycles of resumption of menses (efficacy phase 1). During study phase 2, PF-treated females from study phase 1 contributed additional cycles (6–8) of exposure (efficacy phase 2), and 5 control females who had recovered from medical abortion (after study phase 1 pregnancy) were subsequently treated with 5% PF (with DMPA) and exposed to breeding (efficacy phase 1; *n*=3 six cycles, *n*=2 five cycles).

**Results:**

All females resumed normal menstrual cycles and mating activity after DMPA. During efficacy phase 1, 7/9 (78%) control females became pregnant. In contrast, fewer pregnancies occurred in PF-treated females: 5% PF 0/5 (0%), 5%/doxy 1/5 (20%), 3%/doxy 1/4 (25%) and 3%/BZK 1/4 (25%). During efficacy phase 2, only one additional pregnancy occurred (3%/BZK).

**Conclusions:**

A single transcervical treatment with 5% PF prevented pregnancy in most baboons. Cotreatment with doxycycline or BZK did not improve results.

**Implications:**

Transcervical intrauterine administration of PF resulted in a high rate of tubal occlusion with prevention of pregnancy; refinements are needed to increase the contraceptive rate following a single treatment to near 100%.

## 1 Introduction

Permanent methods of contraception are appropriate for women who have completed their desired family size and who wish never to become pregnant again. Although vasectomy also provides permanent contraception, around the world and in the United States, female procedures predominate [1], [2], [3]. Although the limited uptake of vasectomy may reflect poor acceptability by men, many women prefer female permanent contraception to protect themselves from undesired future pregnancies. While over 95% of vasectomized men in the United States report that they are currently or previously married, over 18% of sterilized women have never been married [4]. Unfortunately, the availability of surgical permanent contraception for women varies widely in many regions, leading to unmet need for services [5], [6]. The widespread use and preference for female methods support the development of nonsurgical options.

We have reported that transcervical administration of polidocanol (hydroxy-polyethoxy-dodecane) foam (PF) results in tubal occlusion in macaques and baboons [7], [8]. In baboons, we observed a dose-dependent histological effect consistent with complete tubal occlusion (complete replacement of epithelium with collagen) confined to the intramural tubal segment in most animals treated with 5% PF [7]. Hormonal suppression with intramuscular depot medroxyprogesterone acetate (DMPA) may improve treatment results with PF; complete occlusion was seen in 4/4 females that received 5% PF followed by DMPA but in only 2/3 that received 5% PF without DMPA.

As 5% PF is higher than the concentration currently FDA approved for vein sclerotherapy (1%), we have performed pilot investigations of adjunctive agents to improve the effectiveness of lower concentrations. We previously reported that the addition of dilute (0.01%) benzalkonium chloride (BZK) (a common preservative) to polidocanol improves the stability of the resulting foam [9]. While unproven, we hypothesize that duration of foam exposure may be associated with greater tissue effects.

Doxycycline is another venous sclerosing agent [10]. While the effects of coadministration of PF and doxycycline are unknown, we further hypothesized that combining these agents could improve the rate of tubal occlusion.

Herein, we conducted a pilot fertility study in baboons to determine whether a single transcervical treatment with PF would prevent pregnancy. We evaluated two concentrations of PF and tested whether the administration of intrauterine doxycycline as a co-sclerosant or BZK would improve the contraceptive outcome. We also evaluated the histologic features induced by these treatments.

## 2 Materials and methods

### 2.1 Animal care

All study procedures were approved by the Southwest National Primate Research Center (SNPRC) Institutional Animal Care and Use Committee. Twenty-two adult female and two adult male baboons (*Papio anubis, Papio anubis/hamadryus* hybrids) of proven fertility were used in this study. Animal husbandry provided by SNPRC is in accord with the National Institutes of Health Guidelines for Care and Use of Laboratory Animals [11]. Menstrual cyclicity was monitored by evaluation of sex skin tumescence and confirmed by measurement of serum estradiol and progesterone as previously described [7]. Observation of a semen plug was considered to be evidence of mating. A blood sample for complete blood count and chemistry panel was obtained prior to treatment and at the end of study.

In an effort to standardize our evaluation of animals, all females not expected to be in the follicular phase on the day of evaluation were pretreated with a combined oral contraceptive (30 mcg ethinyl estradiol/150 mcg levonorgestrel) for 15–21 days and then allowed to have a 6–7-day hormone-free interval prior to treatment to induce a withdrawal bleed.

### 2.2 Clinical evaluation of tubal patency

Females underwent a hysterosalpingogram (HSG) procedure to confirm tubal patency as previously described [7]. Briefly, under transabdominal ultrasound guidance, we passed a small silicone HSG balloon catheter (model J-CHSG-503000; Cook, Bloomington, IN, USA) through the cervix into the uterine cavity. A series of digital radiographs was obtained to evaluate tubal patency (unilateral patent, bilateral patent, bilateral nonpatent) following the infusion of small aliquots (1–3 mL) of radiopaque contrast (Isovue®, iopamidol injection; Bracco Diagnostics, Monroe Township, NJ, USA). At the end of the study, tubal patency was assessed ex vivo by introducing an HSG catheter transcervically and then infusing an indigo carmine saline solution into the uterine cavity under mild pressure observing for spill of dye from the tubal fimbria.

### 2.3 Polidocanol foam and adjunctive treatments

Polidocanol 3% and 5% solutions were prepared by mixing polidocanol stock (Sigma P9641) with sterile physiologic buffered saline. To prepare polidocanol solution containing 0.01% BZK, we added BZK (Sigma-Aldrich 234427) to 3% stock polidocanol solution mixing overnight [9].

We prepared PF using a ratio of 1 mL solution to 4 mL air to generate 5 mL foam [7]. For all PF treatment groups, a total of 20 mL of foam was instilled via the HSG catheter into the uterine cavity over 2–4 min.

Some females received doxycycline (100 mg/mL), prepared by diluting pharmaceutical-grade doxycycline powder (Novaplus, Irving, TX, USA) in sterile saline, as a 1-mL bolus via the HSG catheter following the foam treatment.

All control and PF-treated females received a single dose of DMPA (2.0 mg/kg, im; Pharma & Upjohn [Pfizer], New York, NY, USA) immediately following the HSG examination.

### 2.4 Contraceptive experiment

**Fig. 1:**
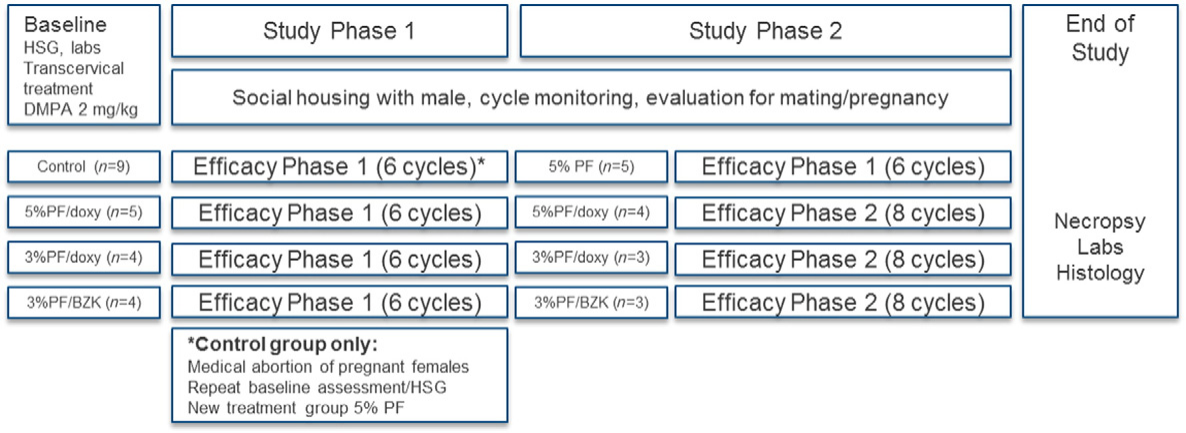
Design of the contraceptive experiment.

The overall design of the contraceptive experiment is illustrated in Fig. 1. In study phase 1, the initial treatment groups were 5% PF followed by doxycycline (5%/doxy), 3% PF plus doxycycline (3%/doxy), 3% PF with 0.01% BZK (3%/BZK) and untreated controls. We defined the first six ovulatory cycles of exposure to the male as efficacy phase 1. Study phase 2 began after completion of this initial efficacy phase. Nonpregnant PF-treated animals remained in the breeding groups for additional cycles of exposure to pregnancy (efficacy phase 2). The control group was disbanded, but a subgroup who had become pregnant and subsequently recovered from medical abortion underwent a repeat HSG followed by treatment with 5% PF (no doxycycline) followed by DMPA. Following resumption of cycles, these females (5% PF) were reintroduced to the breeding groups and contributed efficacy phase 1 data.

#### 2.4.1 Establishment of breeding groups

Following recovery, females were socially housed in two groups that contained a mix of control and PF-treated animals. After resumption of menstrual cyclicity, an adult male of proven fertility was introduced to each group.

#### 2.4.2 Verification of ovulatory cycles and mating

At least 3 days per week, females underwent visual evaluation of sex skin tumescence to assess menstrual cycle phase and evidence of mating (semen plug) [12].

#### 2.4.3 Evaluation and management of pregnancy

Females that failed to exhibit an expected menses underwent ultrasound evaluation on approximately cycle day 35. A diagnosis of pregnancy was confirmed by the observation of fetal heart activity. In cases where ultrasound evidence was inconclusive, a serum sample was obtained to assess for monkey chorionic gonadotropin (mCG) by radioimmunoassay using antiserum H-26, previously established to determine mCG concentrations in blood, urine and cells, in the Endocrine Technologies Support Core at the Oregon National Primate Research Center [13]. The standard curve ranged from 0.005 to 10 ng/tube; the detection limit of the assay was 0.005–2 ng/tube. Intra- and interassay variations for this assay were less than 10% and 15%, respectively.

Pregnant control females underwent medical abortion with mifepristone (Danco Pharmaceuticals, New York, NY, USA) 200 mg orally followed by misoprostol (Gavis Pharmaceuticals, Somerset, NJ, USA) 400–800 mcg vaginally or buccally 24 h later. Animals were singly housed during the procedure and observed for signs and symptoms of expulsion. If expulsion did not occur within 24 h, the misoprostol dose was repeated. Transabdominal ultrasound was performed after 1 week to monitor treatment success. To assess possible adverse effects of polidocanol on pregnancy, PF-treated females underwent induction of labor at term. The fetus was euthanized to allow for pathologic evaluation.

### 2.5 Gross and histologic evaluations

Animals underwent necropsy by veterinary pathologists at the SNPRC Pathology Services Unit according to their standard protocol for collection of the reproductive tract and evaluation of other organs. The uterus and cervix, upper vagina, bilateral tubes and ovaries were extirpated as a single specimen and prepared for histological evaluation as previously described [7]. Representative sections through both corneal areas were further evaluated for histologic evidence of tubal occlusion (an obliterated lumen in at least a focal 10× objective field). Immunostaining for cytokeratin, using routine methods in our laboratory [14], was used to detect the presence of tubal epithelium when results were unclear. We also performed immunostaining for collagen 3, using our previously published methods [15], to better characterize the treatment effect.

### 2.6 Data analysis

The primary outcome was the frequency of pregnancy within treatment groups during efficacy phase 1. Based upon previous experience of the breeding colony at SNPRC, we expected that 80% of control females would become pregnant in 6 months (unpublished data on file, SNPRC). Although the sample size in the treatment groups is insufficient for robust statistical analysis, we hypothesized that zero pregnancies would occur following a successful treatment.

We used the Wilson method (http://epitools.ausvet.com.au) to calculate 95% confidence intervals (95% CIs) around the proportion of pregnancies observed in treated and control females. Secondary outcomes included frequency of pregnancy during efficacy phase 2 (5%/doxy, 3%/doxy and 3%/BZK groups only), histologic evaluation of the intramural tube and safety.

## 3 Results

### 3.1 Clinical assessment of tubal patency and treatment groups

At the initial HSG examination, bilateral tubal patency was confirmed in 17/22 females. Bilateral patency was confirmed in all of the females treated with PF; five were treated with 5% PF/doxy, four received 3% PF/doxy, and four received 3% PF/BZK. The control group consisted of nine females that did not receive intrauterine PF; HSG confirmed bilateral patency in only four. All 22 females resumed regular menstrual cycles 6–10 weeks after receiving DMPA, and evidence of mating was recorded in most cycles (see supplemental material and Supplemental Table 1). During study phase 2, five females were treated with 5% PF (no doxycycline); bilateral patency was confirmed in only four.

### 3.2 Contraceptive effect

**Fig. 2:**
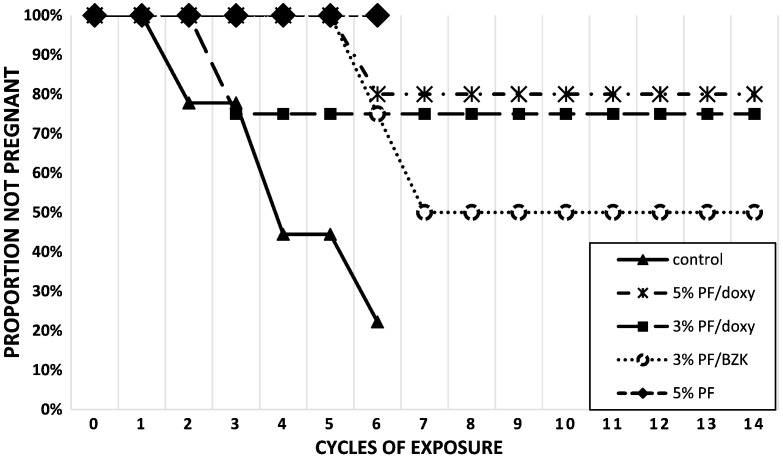
Time to pregnancy for animals followed during the study. Control group and 5% PF group followed for only six cycles.

During efficacy phase 1 (6 cycles), 7/9 [78% (95% CI, 45%–94%)] control females became pregnant; 1 pregnancy each occurred in the 5% PF/doxy [1/5, 20% (4%–62%)], 3% PF/doxy [1/4, 25% (5%–70%)] and 3% PF/BZK [1/4, 25% (5%–70%)] treatment groups (study phase 1); and no pregnancies occurred in the 5% PF [0/5, 0% (0%–52%)] group (study phase 2) (Table 1 and Fig. 2). In the 5% PF group, three females contributed six cycles of exposure, and two contributed only five cycles.

During efficacy phase 2, one additional treatment failure was seen in the 3%/BZK group, bringing the cumulative failure to 50% (15%–85%). No further pregnancies occurred in females treated with 5%/doxy or 3%/doxy.

### 3.3 Clinical safety

No treatment-related adverse events occurred during the study. One pregnant control female died of group B streptococcal sepsis prior to undergoing medication abortion. All of the pregnancies that occurred in PF-treated animals were intrauterine and progressed uncomplicated to term. We performed a feticidal injection prior to induction of labor at 165 days of gestational age; vaginal delivery occurred in three, and one underwent cesarean section for failed induction. The fetuses were of normal size and appeared grossly normal (see Supplemental Table 3) [16].

PF-treated females underwent necropsy either at 4–6 weeks after completion of term pregnancy or after the end of study phase 2 in females that did not become pregnant (8–17 months after PF treatment). One of the control females underwent necropsy immediately after death due to sepsis (see above) and three others 2 months following the completion of study phase 1. Routine general pathologic survey and histologic samples (e.g., heart, liver, kidneys and brain) revealed only minor background findings common in the baboon population at SNPRC (see Supplemental Table 2 for a summary of pathologic findings). Since PF could potentially have adverse intraperitoneal effects, we specifically evaluated for the presence of pelvic adhesions. Significant pelvic adhesions involving the anterior and posterior cul-de-sac and bilateral fallopian tubes were noted in only one female (5%/doxy group). Mild adhesions not involving the tubal fimbriae or ovary were noted in two 5% PF-treated females (small bowel mesentery to right tube, anterior cul-de-sac peritoneum to right round ligament) and in one treated with 3%/BZK (omentum to fundus). The results of complete blood count and serum chemistry panels obtained at end of study remained within the normal range for all assays, with no clinically significant changes from baseline.

### 3.4 Clinical and histologic assessment of tubal patency at end of study

At the end of study, all females treated with PF that did not become pregnant showed evidence of bilateral tubal occlusion on the ex vivo dye test, except one animal treated with 3%/BZK (Table 2).

**Fig. 3:**
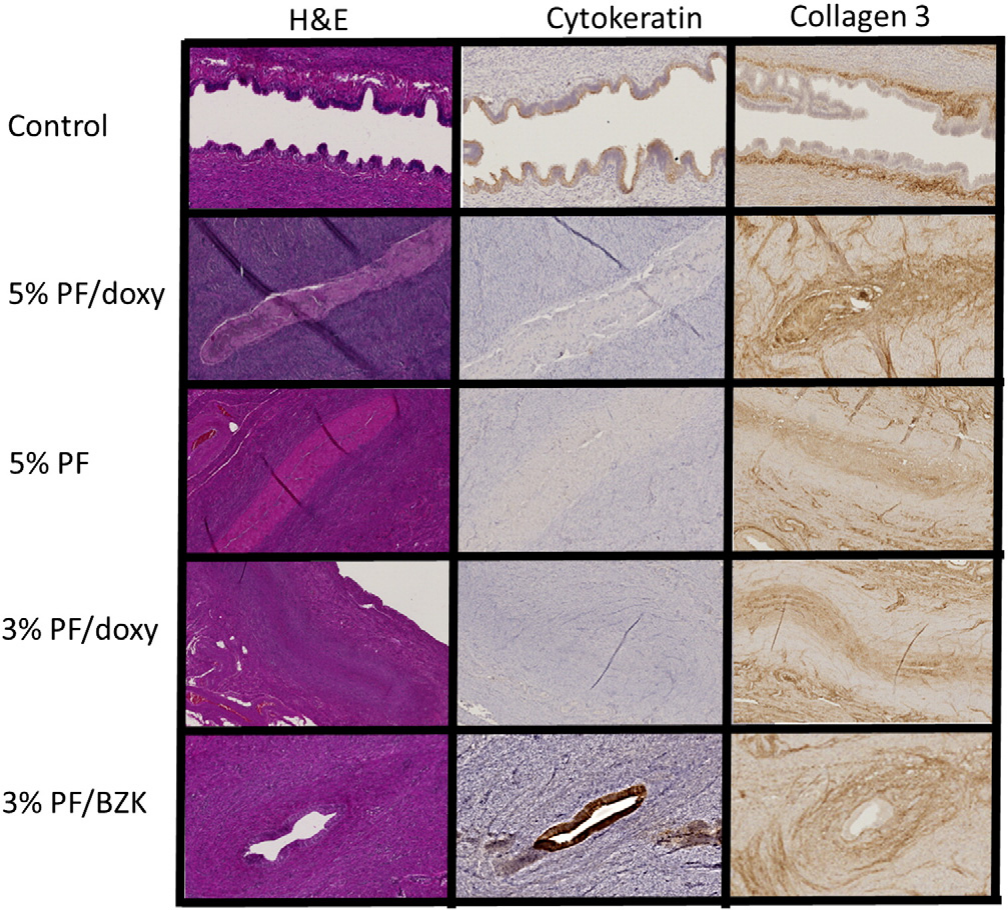
Histologic features of the intramural fallopian tube after no treatment (control), 5% polidocanol foam with (5% PF/doxy) and without (5% PF) doxycycline, and 3% PF with doxycycline (3% PF/doxy) or BZK (3% PF/BZK). Treatment with PF resulted in a loss of tubal epithelium and obliteration of the lumen. Absence of staining for cytokeratin, an epithelial cell marker, indicates loss of epithelial cells. Staining for collagen 3 demonstrates replacement of epithelium with connective tissue consistent with a permanent scar. Changes were more prominent in animals treated with 5% PF and less extensive in those treated with 3% PF/BZK.

Females that became pregnant showed normal histologic features (similar to untreated control animals) in one or both tubes (Fig. 3). In contrast, histologic evaluation of animals that failed to conceive generally revealed bilateral obliteration of the fallopian tube lumen with collagen replacement of epithelium confined to the intramural tube as we have previously described [7] (Fig. 3). Exceptions included unilateral blockade with one normal tube in both of the females treated with 3% PF/BZK and in one treated with 3% PF/doxy. One of the females treated with 5% PF also showed bilateral normal fallopian tubes. This animal had two HSG exams in study phase 1 that both showed tubal obstruction prior to control group assignment. She conceived after five cycles and underwent an uncomplicated medical abortion. During study phase 2, a third HSG prior to treatment with 5% PF again demonstrated bilateral obstruction. Given the lack of patency observed during the pre-PF HSG, it is highly likely that the intramural tube was not adequately treated.

When tubal occlusion developed, the histologic changes were similar with all treatments. However, treatment with 5% PF (with or without doxycycline) resulted in more extensive effects than 3% PF (Fig. 3). Furthermore, the two females treated with 3% PF/BZK that did not become pregnant showed only unilateral occlusion, with only focal areas of luminal obstruction and nests of normal epithelium between these areas.

## 4 Discussion

This is the first study to demonstrate that a single transcervical administration of polidocanol foam can prevent pregnancy in primates. We have reported that treatment with PF results in histologic features of tubal occlusion in rhesus monkeys [8] and baboons [7]. Herein we show that tubal occlusion is lasting and thereby provides permanent contraception. We report that, up to 16 months after PF treatment, the histologic features of tubal occlusion confined to the intramural zone persist and that females with bilateral occlusion fail to conceive.

While encouraging, our results are limited by the small number of females in each group, which makes comparisons between adjunctive agents difficult. Although the control group experienced the expected number of pregnancies and fewer pregnancies occurred in females treated with PF, failures did occur with all treatments except 5% PF. Since the length of exposure to pregnancy was shorter for the 5% PF group than other treatments, we interpret these results with caution.

In this study, the presence of histologic tubal occlusion in the 5% PF treated animals was similar to our previous reports [7]. We decided to test more than one approach to PF treatment in order to learn more about potential strategies to reduce dose and improve efficacy. In our prior study with baboons, we found that administration of DMPA following PF treatment improved the rate of complete tubal occlusion and that 5% PF was superior to 1% [7]. However, only 1% polidocanol is currently approved for human use in the United States. Similar to published results in macaques [10], we found that transcervical administration of doxycycline results in tubal obstruction in baboons but that more than one treatment is needed (unpublished results). Therefore, we hypothesized that intrauterine administration of doxycycline after PF might provide a second “hit” and allow us to reduce the concentration of PF. Unfortunately, this combination failed to increase the rate of success. It is possible that the administration of doxycycline solution flushed PF from the intramural tube, reducing the treatment effect in some animals and allowing healing and normal tubal function. We also tested whether BZK could improve PF stability and lower the concentration needed to create tubal blockade. This cotreatment also failed to affect the rate of contraception.

While the use of the nonhuman primate model provides a direct translational link to human medicine, there are limitations. A major weakness of our study is that the limitation of animal numbers resulted in very small and unbalanced treatment groups, and we are missing several important control groups including 3% PF with no adjunctive treatment and no additional control cycles of treatment following successful medical abortion. A further weakness of our study is that small animal numbers prevent full quantified evaluation of the histology, and our impressions are subjective.

Although 5% polidocanol exceeds the 1% strength currently approved for vein treatments, this higher concentration may be required for adequate sclerosis of the tubal epithelium and subepithelial tissue to promote collagen deposition and prevent reepithelialization. To address the regulatory challenge for approval of this concentration, we are currently investigating modifications of the delivery system to reduce the total volume of foam delivered in order to keep the milligram amount of polidocanol administered within the single-treatment approved range (19.5 mg for Varithena™).

The risk of ectopic pregnancy is increased following tubal ligation procedures for permanent contraception [17]. Hieu et al. [18] performed a case–control study of ectopic pregnancy in Vietnam and found the risk for quinacrine sterilization (0.26/1000 woman-years) to be equivalent to that of surgical sterilization (0.42/1000 woman-years) and significantly lower than that in women using no method of contraception. Inadequate treatment with polidocanol foam could result in tubal damage and increase the risk of ectopic pregnancy. Although ectopic pregnancy has been reported in nonhuman primates, it is extremely rare, so assessment of this risk will await human clinical trials.

Our results support additional investigations of polidocanol foam for permanent contraception. We are currently evaluating strategies to improve the delivery of PF to improve efficacy at a lower dose at the Oregon Permanent Contraception Research Center (https://www.ohsu.edu/OPERM/).

The following are the supplementary data related to this article.

Supplemental Appendix Image 1

Supplemental Table 1 Cycle characteristics during study phase 1 and study phase 2. Supplemental Table 1

Supplemental Table 2 Gross and general pathologic findings recorded at end of study necropsy. Supplemental Table 2

Supplemental Table 3 Gross and final pathologic findings recorded for fetuses delivered from PF-treated females. Supplemental Table 3

## Figures and Tables

**Table 1:** Number of pregnancies that occurred in baboons during the study

		Number (%) pregnant		
	n	Efficacy phase 1	Efficacy phase 2	Cumulative
Study phase 1				
Control group	9	7 (78%)	–	7/9 (78%)^a^
5% PF+doxy	5	1 (20%)	–	
3% PF+doxy	4	1 (25%)	–	
3% PF+BZK	4	1 (25%)	–	

Study phase 2				
5% PF	5	0 (0%)	–	0/5 (0%)^a^
5% PF+doxy	4	–	0 (0%)	1/5 (20%)
3% PF+doxy	3	–	0 (0%)	1/4 (25%)
3% PF+BZK	3	–	1 (33%)	2/4 (50%)

a Control and 5% PF groups completed efficacy phase 1 only.

**Table 2:** Evaluation of tubal patency at end of study in animals that did not become pregnant

	Patency assessment					
Treatment	Ex vivo dye test			Histologic assessment		
		Occlusion			Occlusion	
	Open	Unilateral	Bilateral	Open	Unilateral	Bilateral
5% PF/doxy (*n*=4)	0	0	4	0	0	4
5% PF (*n*=5)	0	0	5	1	0	4
3% PF/doxy (*n*=3)	0	0	3	0	1	2
3% PF/BZK (*n*=2)	0	1	1	0	2	0
