# Supplementary material for: Transcervical administration of polidocanol foam prevents pregnancy in female baboons
Source: Contraception. 2016 Nov;94(5):527–33. doi: 10.1016/j.contraception.2016.07.008 (PMC5083254; doi:10.1016/j.contraception.2016.07.008)
Supplement: Supplementary file 1 — Supplemental Appendix [file mmc1.docx]

**Supplemental Appendix.**

Females received depot medroxyprogesterone acetate 2mg/kg following hysterosalpingogram (controls) or treatment with polidocanol foam. Females were then socially housed, and a fertile male was introduced 2-3 months after treatment and remained with the females until end of study. The first month of male introduction after treatment was cycle 1. Menstrual cyclicity was determined by observation of sex skin tumescence at least 3 days per week, and mating was confirmed by observation of a semen plug. All of the non-pregnant females showed evidence of mating with a semen plug at least once during the phase 1 and phase 2 study interval (applicable to treatment groups 5% PF + doxy, 3% PF + doxy, and 3% PF + BZK only). Three of the 7 pregnant control females did not show a semen plug prior to the diagnosis of pregnancy. This provides evidence that the failed observation of a semen plug does not rule out the possibility of mating during a normal cycles. A semen plug was never observed in females that were non-receptive by cycle stage. The mean number of semen plugs observed in females that became pregnant during Study Phase 1 was 1.54 (17 plugs in 11 females) compared to 2.93 (44 in 15) observed in non-pregnant females. It would be expected that a contraceptive effect would be associated with more coital events that do not result in pregnancy.
