# Supplementary material for: Transcervical administration of polidocanol foam prevents pregnancy in female baboons
Source: Contraception. 2016 Nov;94(5):527–33. doi: 10.1016/j.contraception.2016.07.008 (PMC5083254; doi:10.1016/j.contraception.2016.07.008)
Supplement: Supplemental Table 1 — Cycle characteristics during study phase 1 and study phase 2. [file mmc2.docx]

**Supplemental Table 1.**

|  | **Study Phase 1** | | | | | | | **Study Phase 2** | | | | | | |
| --- | --- | --- | --- | --- | --- | --- | --- | --- | --- | --- | --- | --- | --- | --- |
| Animal ID | **Cycle 1** | **Cycle 2** | **Cycle 3** | **Cycle 4** | **Cycle 5** | **Cycle 6** | **Cycle 7** | **Cycle 8** | **Cycle 9** | **Cycle 10** | **Cycle 11** | **Cycle 12** | **Cycle 13** | **Cycle 14** |
| **Control** | | | | | | | | | | | | | | |
| 1**29 | yes | yes | yes | yes | yes | PREG | MAB | - | - | - | - | - | - | - |
| 27**8 | yes | PREG | MAB | no | yes | yes | yes | - | - | - | - | - | - | - |
| **1**57** | yes | yes | yes | yes | yes | yes | yes | - | - | - | - | - | - | - |
| 2**34 | yes | ?? | yes | PREG | SAB | no | yes | - | - | - | - | - | - | - |
| 2***8 | yes | PREG | MAB | no | no | yes | yes | - | - | - | - | - | - | - |
| 19**0 | yes | yes | yes | PREG | MAB | no | no | - | - | - | - | - | - | - |
| 1**67 | no | no | yes | yes | yes | PREG | MAB | - | - | - | - | - | - | - |
| 2**96 | no | no | yes | PREG | SAB | - | - | - | - | - | - | - | - | - |
| 2**88 | yes | yes | yes | yes | yes | yes | yes | - | - | - | - | - | - | - |
| **5% polidocanol foam + doxycycline** | | | | | | | | | | | | | | |
| 2**90 | yes | yes | yes | yes | yes | yes | yes | yes | yes | yes | yes | yes | yes | yes |
| 2***7 | yes | yes | yes | yes | yes | yes | PREG | PREG | PREG | PREG | PREG | PREG | - | - |
| 2**40 | yes | yes | yes | yes | yes | yes | yes | yes | yes | yes | yes | yes | yes | yes |
| 2**67 | yes | yes | yes | yes | yes | yes | yes | yes | yes | yes | yes | yes | yes | yes |
| 2**87 | yes | yes | yes | yes | yes | yes | yes | yes | no | yes | yes | no | no | no |
| **3% polidocanol foam + doxycycline** | | | | | | | | | | | | | | |
| 2**81 | yes | yes | yes | PREG | PREG | PREG | PREG | PREG | PREG | - | - | - | - | - |
| 1**88 | yes | yes | yes | yes | yes | yes | yes | yes | no | no | yes | yes | yes | yes |
| 1**20 | yes | yes | yes | yes | yes | yes | yes | yes | yes | yes | yes | yes | yes | yes |
| 1**55 | no | yes | yes | yes | yes | yes | yes | yes | yes | yes | yes | yes | yes | yes |
| **3% polidocanol foam + BZK** | | | | | | | | | | | | | | |
| 1**60 | no | yes | yes | yes | yes | yes | yes | yes | yes | no | yes | yes | yes | yes |
| 1**39 | no | yes | yes | yes | yes | yes | PREG | PREG | PREG | PREG | PREG | PREG | - | - |
| 2**64 | yes | yes | yes | yes | yes | yes | yes | PREG | PREG | PREG | PREG | PREG | PREG | - |
| 2**24 | no | no | yes | yes | yes | yes | yes | yes | yes | yes | yes | yes | yes | yes |
| **5% polidocanol foam** | | | | | | | | | | | | | | |
| 1**29 | yes | yes | yes | yes | yes | yes | - | - | - | - | - | - | - | - |
| 19**0 | no | yes | yes | yes | yes | yes | - | - | - | - | - | - | - | - |
| 2**34 | yes | yes | yes | yes | yes | yes | - | - | - | - | - | - | - | - |
| 2***8 | no | yes | yes | yes | yes | yes | - | - | - | - | - | - | - | - |
| 27**8 | yes | yes | yes | yes | yes | yes | - | - | - | - | - | - | - | - |
