# Supplementary material for: Transcervical administration of polidocanol foam prevents pregnancy in female baboons
Source: Contraception. 2016 Nov;94(5):527–33. doi: 10.1016/j.contraception.2016.07.008 (PMC5083254; doi:10.1016/j.contraception.2016.07.008)
Supplement: Supplemental Table 2 — Gross and general pathologic findings recorded at end of study necropsy. [file mmc3.docx]

**Supplemental Table 2**

| **Animal ID/Treatment** | **Gross pelvic findings** | **Pathology final report** |
| --- | --- | --- |
| **5% Polidocanol foam (PF) + doxy** |  |  |
| **2**90** | 0 | no significant findings |
|  |  | normal iliac vessels |
| **2***7** | 0 | no significant findings |
|  |  | normal iliac vessels |
| **2**40** | 0 | parathyroid cyst (not related) |
|  |  | area of fibrosis, left iliac vein |
| **2**67** | 5 | no significant findings |
|  |  | normal iliac vessels |
| **2**87** | 0 | pituitary cyst (not related)  chronic inflammatory bowel (not related) |
|  |  | normal iliac vessels |
|  |  |  |
| **3% PF + Doxy** |  |  |
| **2**81** | 0 | thyroid hyperplasia (not related)  normal iliac vessels |
| **1**88** | 0 | no significant findings |
|  |  | normal iliac vessels |
|  |  | nephritis (not related) |
| **1**20** | 0 | no significant findings |
|  |  | normal iliac vessels |
| **1**55** | 0 | no significant lesions |
|  |  | normal iliac vessels |
|  |  |  |
| **3% PF + BZK** |  |  |
| **1**60** | 0 | no significant findings |
|  |  | normal iliac vessels |
| **1**39** | 0 | anthracosilicosis (not related) |
|  |  | normal iliac vessels |
| **2**64** | 1 | thyroid hyperplasia (not related)  anthracosilicosis (not related) |
|  |  | normal iliac vessels |
| **2**24** | 0 | nephritis (not related) |
|  |  | normal iliac vessels |
|  |  |  |
| **Control + 5% PF** |  |  |
| **1**29** | 0 | no significant findings |
|  |  | normal iliac vessels |
|  |  |  |
| **19**0** | 0 | epicardial fibrosis (not related) |
|  |  | normal iliac vessels |
| **2**34** | 0 | no significant findings |
|  |  | normal iliac vessels |
| **2***8** | 0 | no significant findings |
|  |  | normal iliac vessels |
| **27**8** | 1 | no significant findings |
|  |  | normal iliac vessels |
| **Control + 5%PF NO DMPA** |  |  |
| **1**57** | 0 | peripyelitis (not related) |
|  |  | normal iliac vessels |
| **1**67** | 0 | no significant findings |
|  |  | normal iliac vessels |
| 2**88 | 1 | no significant findings |
|  |  | normal iliac vessels |
| Control only |  |  |
| 2**96 | 0 | findings consistent with hepatic and renal failure secondary to sepsis |
|  |  | normal iliac vessels |
