# Supplementary material for: Transcervical administration of polidocanol foam prevents pregnancy in female baboons
Source: Contraception. 2016 Nov;94(5):527–33. doi: 10.1016/j.contraception.2016.07.008 (PMC5083254; doi:10.1016/j.contraception.2016.07.008)
Supplement: Supplemental Table 3 — Gross and final pathologic findings recorded for fetuses delivered from PF-treated females. [file mmc4.docx]

**Supplemental Table 3**

| **Animal ID/Treatment** | **Gross findings** | **Pathology final report** |
| --- | --- | --- |
| **5% Polidocanol foam (PF) + doxy** |  |  |
| **3**84** | Normal morphology, no significant gross findings  Weight = 1010 grams | No significant gross or internal lesions  Moderate to marked autolysis |
| **3% PF + Doxy** |  |  |
| **3**06** | Adequate muscle mass, hydration and adipose tissue  Weight = 830 grams | No significant gross or internal lesions  Marked autolysis |
| **3% PF + BZK** |  |  |
| **3**18** | Normal morphology, marked autolysis  Weight not recorded | Chorioamnionitis  Extensive autolysis |
|  |  |  |
| 3**63 | Normal morphology, no significant gross lesions  Weight = 1100 grams | No significant gross or internal lesions  Moderate to marked autolysis |
